# Ultra-processed foods consumption among children and associated socioeconomic and demographic factors

**DOI:** 10.31744/einstein_journal/2021AO5554

**Published:** 2021-08-25

**Authors:** Eveline Costa Cainelli, Brunna Verna Castro Gondinho, Danielle da Costa Palacio, Daniele Boina de Oliveira, Roberta Andrade Reis, Karine Laura Cortellazzi, Luciane Miranda Guerra, Denise de Fátima Barros Cavalcante, Antonio Carlos Pereira, Jaqueline Vilela Bulgareli

**Affiliations:** 1 Faculdade de Odontologia de Piracicaba Universidade Estadual de Campinas PiracicabaSP Brazil Faculdade de Odontologia de Piracicaba, Universidade Estadual de Campinas, Piracicaba, SP, Brazil.; 2 Hospital Israelita Albert Einstein São PauloSP Brazil Hospital Israelita Albert Einstein, São Paulo, SP, Brazil.

**Keywords:** Infant nutritional physiological phenomena, Infant, Infant nutrition, Family health strategy

## Abstract

**Objective:**

To evaluate the consumption of ultra-processed foods among children, and to investigate associations with socioeconomic and demographic factors.

**Methods:**

An analytical cross-sectional study with 599 children aged 6 months to 2 years, and listed as users of Family Health Units, in a medium-size city. Mothers were approached at home by researchers and community health workers from the Family Health Units, for data collection. Two questionnaires were used: the socioeconomic and demographic questionnaire, and the form *Sistema de Vigilância Alimentar e Nutricional* of *Ministério da Saúde do Brasil* , for children aged 6 months to 2 years. Ultra-processed food consumption and socioeconomic and demographic factors were defined as dependent and independent variables, respectively. Multiple regression analysis with a significance level of 5% was used to test associations between ultra-processed food consumption and socioeconomic and demographic variables.

**Results:**

Ultra-processed food consumption was associated with child age between 1 and 2 years (OR=3.89; 95%CI: 2.32-6.50 and OR=3.33; 95%CI: 2.00-5.56, respectively), number of people living in the same household (OR=1.94; 95%CI: 1.23-3.05), and recipients of government benefits (OR=1.88; 95%CI: 1.15-3.04).

**Conclusion:**

Ultra-processed food consumption among children undergoing complementary feeding may be influenced by socioeconomic and demographic factors.

## INTRODUCTION

The first 1,000 days of life define the cycle from fertilization to the first 2 years of life and have a direct impact on human development. From the nutritional standpoint, supplementation during pregnancy, breast feeding and complementary feeding are three effective strategies applicable to this period.^(1)^

The complementary feeding phase starts in the sixth month of life. In this phase, purees or mashed foods from different groups are slowly and gradually introduced along with breast milk.^(2)^

Increased gastrointestinal tolerance and ability to absorb nutrients as from the age of 6 months allows physical and physiological adaptation of children to heterogeneous diets comprising foods with different consistency and texture. It is important to offer a wide variety of foods and to avoid foods with high sugar, saturated and trans fat content, or containing additives and coloring agents to provide the child with all necessary nutrients while promoting dietary habits and preventing dietary monotony.^(3)^

Ultra-processed foods (UPF) include treats, artificial sweetener and sugar-sweetened beverages, cold meats and several other new products on offer every year. These are industrialized products made with substances extracted from foods or synthesized in laboratories from organic materials, such as oil and coal.^(4,5)^

Scientific evidence suggests the replacement of home cooked and *in natura* foods with UPF leads to excess body weight, chronic noncommunicable diseases, and specific nutritional deficiencies in childhood, with potential impacts on adult life.^(6)^ These dietary changes have been observed across all socioeconomic brackets, including the low income stratum.^(7)^

In Brazil, excess body weight and obesity are a matter of concern, particularly in children.^(8)^

Ultra-processed foods should not be included in complementary feeding due to their potentially negative effects on child overall and oral health.^(9,10)^ The investigation of UPF consumption in this phase and potential associations with socioeconomic and demographic factors may provide a comprehensive view of food choices made by parents or responsible persons. These data may help health professionals and managers to rethink actions and policies aimed to improve child feeding.

## OBJECTIVE

To examine the consumption of ultra-processed foods among children and to investigate associations with socioeconomic and demographic factors.

## METHODS

### Ethical considerations

In compliance with resolution 466/2012 issued by the National Health Council of the Ministry of Health, this study was approved by the Research Ethics Committee of *Faculdade de Odontologia de Piracicaba, Universidade Estadual de Campinas* (UNICAMP), opinion No. 1.852.022, CAAE: 61502116.6.0000.5418. All participants signed an Informed Consent Term (ICF).

### Settings, population and type of study

This study was carried out in Piracicaba (SP), a medium-size city with an estimated population of 404,142 thousand inhabitants, land area of 1,378.069km^2^ and population density of 264.47 inhabitants/km^2^. This region comprises 122 facilities of the Unified Health System (SUS - *Sistema Único de Saúde*).^(11)^

This is an analytical, cross-sectional study, based on a target population of children aged 6 months to 2 years listed as users of municipal Family Health Units (FHU), totaling up 1,169 children from January to April 2016, as per the Municipal Health Department.

### Selection and sample

Sample size was calculated using Epi Info™ 7 with a 95% confidence interval (95%CI). Assuming a power of 80%, a rate of not exposed to exposed of 1, a percentage of response of 73% in the not exposed group, and odds ratio (OR) of 1.8, it was determined that a minimum sample size of 582 randomly selected individuals would be required.

This sample comprised mothers who were present on the day of data collection. The following selection criteria were applied: age between 18 and 50 years, children aged 6 months to 2 years, and ability to describe the child’s diet the day before.

### Study design

This sample comprised 599 children listed as users of municipal FHUs from February to July 2017. Data collection date and time were scheduled by FHUs managers via telephone call.

Mothers were approached at home by researchers and a Community Health Worker. On this occasion, mothers were duly informed about research objectives and those who agreed to participate signed an ICF, and answered questions about socioeconomic and demographic factors and the child’s diet the day before. Mothers were allowed to clear up doubts about child nutrition.

### Data collection instrument

Socioeconomic data were collected using the instrument designed by Meneghim et al.,^(10)^(Appendix 1) plus questions about demographic characteristics (age, marital status, number of children, whether the mother works outside the home, who is the head of the family, whether the household has TV and/or internet and whether they receive government benefits).

**Appendix 1:** Questionnarie based on Meneghim et al.,(10)

Socioeconomic and demographic questionnaire									
Name of mother: _____________________________								Age: _______	
Name of child: ____________________________								Age: _______	
Sex of child:	a) F	b) M		Date of child birth: ___-___-___					
1. Marital status:	a) Single	b) Married		c) De facto relationship			d) Separated	e) Divorced	f) Widower
2. Number of children (excluding this child):			a) 1		b) 2	c) 3	d) 4	e) More than 5	
3. Economic status of family (monthly household income in wages):									
a) R$ 937.00 or less	b) R$ 937.00 to R$ 1,874.00								
c) R$ 1,874.00 to R$ 2,811.00	d) R$ 2,811.00 to R$ 4,685.00								
e) R$ 4,685.00 to R$ 6,559.00	f) R$ 6,559/00 to R$ 10,307.00								
g) R$ 10,307.00 or more									
4. Number of people living in the same house:									
a) 2 or less				b) 3 people				c) 4 people	
d) 5 people				e) 6 people				f) More than 6	
5. Your home is:									
a) Owned and paid off				b) Owned with mortgage payments due					
c) Leased by parents or relativces				d) Leased in exchange for work					
e) Rented				f) Granted tenancy due to lack of a place to live					
6. What is your level of education:									
a) Illiterate				b) Literate					
c) Incomplete primary education				d) Complete primary education					
e) Incomplete junior school				f) Complete junior school					
g) Incomplete secondary education				h) Complete secondary education					
i) Incomplete higher education				j) Complete higher education					
7. Who is the head of the family?									
a) Mother				b) Father					
8. What is the head of the family’s profession (inform even if unemployed)? _________________									
9. Do you work outside the home?				a) Yes				b) No	
10. If yes, who takes care of the child when you are at work? _____________________________									
11. The household has?	a) Television			b) Internet				c) Television and internet	
12. Do you receive government benefits (inform)?				a) Yes __________________________				b) No	

This study employed the form *Marcadores de Consumo Alimentar* developed by *Sistema de Vigilância Alimentar e Nutricional* (Sisvan) of *Ministério da Saúde* for children aged 6 months to 2 years, based on a document published by the World Health Organization (WHO)^(12)^ (Appendix 2).

**Appendix 2:** Food consumption form for children aged 6 months to 2 years

Tick all questions:	( ) Yes	( ) No	( ) Don’t know					
Was the child breastfed yesterday?						( ) Yes	( ) No	( ) Don’t know
Did the child eat whole, chopped or mashed fruit yesterday?						( ) Yes	( ) No	( ) Don’t know
If yes, how often?					( ) Once	( ) Twice	( ) 3 times or more	( ) Don’t know
Did the child eat salty foods yesterday (cooked, puree or soup)?						( ) Yes	( ) No	( ) Don’t know
If yes, how often?					( ) Once	( ) Twice	( ) 3 times or more	( ) Don’t know
If yes, How was this food offered?					( ) Chopped	( ) Mashed	( ) Sieved	( ) Liquefied
Milk other than breast milk						( ) Yes	( ) No	( ) Don’t know
Porridge with milk						( ) Yes	( ) No	( ) Don’t know
Yogurt						( ) Yes	( ) No	( ) Don’t know
Legumes (other than water yam, taro, potatoes, cassava)						( ) Yes	( ) No	( ) Don’t know
Orange-colored vegetable or fruit (papaya, pumpkin) or dark green vegetables (kale, spinach, scarole)						( ) Yes	( ) No	( ) Don’t know
Leafy greens (lettuce, Chinese leaves, cabbage).						( ) Yes	( ) No	( ) Don’t know
Meat, offal or egg						( ) Yes	( ) No	( ) Don’t know
Liver						( ) Yes	( ) No	( ) Don’t know
Beans						( ) Yes	( ) No	( ) Don’t know
Rice, potatoes, taro, cassava, flour or pasta (other than instant)						( ) Yes	( ) No	( ) Don’t know
Hamburger or cold meats: sausage, ham, salami						( ) Yes	( ) No	( ) Don’t know
Sweetened beverages (powdered juice, soda, processed juice, sugar-sweetened fruit juice, chocolate milk, thickener)						( ) Yes	( ) No	( ) Don’t know
Instant pasta, chips or salty biscuits						( ) Yes	( ) No	( ) Don’t know
Cream-filled biscuits or treats (jelly, candy, lollypop and chewing gum)						( ) Yes	( ) No	( ) Don’t know

This form includes questions about food quality and timing of introduction, identification of risk, or protection against nutritional deficiencies, and occurrence of excess body weight. It comprises 20 closed-ended questions with the following answer alternatives: “yes”, “no” or “do not know”. Child sex and age were included. Answers to all 20 questions included in the form were collected. However, only the UPF category was used in this study.

### Study variables

Ultra-processed food consumption (yes or no) was defined as the dependent variable in this study. Ultra-processed foods were defined according to the form, as follows: hamburger and/or cold meats (ham, mortadela, salami and bologna or other sausages); sweetened beverages (soda, processed fruit juice, powdered juice, processed coconut water, guarana or redcurrant syrup, and sugar-sweetened fruit juice); instant pasta, chips or salty biscuits, cream-filled biscuits, sweets or treats.

Independent variables were age, marital status, mother´s number of children, household income, number of people living in the household, home ownership, maternal level of education, and whether the household had TV and/or internet (dichotomized by the median). Child sex was described as female or male. The head of the family was described as mother or father. Child age was categorized as 6 months to 1 year, 1 year to 1 year and 6 months, and 1 year and 6 months to 2 years, as suggested in the form.^(13)^

### Data analysis

Associations between UPF consumption and independent variables were investigated using multiple logistic regression. Variables achieving p≤0.20 in crude analysis were tested in the multiple logistic regression model. Variables that remained associated with UPF consumption (p≤0.05) following adjustments for all other variables included in the analysis were retained in the model. Odds ratios and respective 95%CI were estimated. The level of significance was set at 5%. Statistical tests were performed using SAS software, version 9.4 (SAS Institute Inc., Cary, NC, United States, release 9.4, 2010).

## RESULTS

Table 1 shows distributions of frequency of UPF consumption according to study variables. Ultra-processed food consumption was detected in 79.4% of children. Of these, 50.7% were females, 35.4% were aged 6 months to 1 year, 33.9% were aged 1 to 1 year and 6 months, and 30.7% were aged 1 year and 6 months to 2 years.

**Table 1:** Ultra-processed food consumption according to study variables

Variable	Ultra-processed food consumption		
	Total	Yes	No
Maternal age, years			
≤27	312 (52.1)	251 (80.45)	61 (19.55)
>27	287 (47.9)	225 (78.4)	62 (21.6)
Maternal marital status			
Single	102 (17.2)	89 (87.25)	13 (12.75)
Married/others	492 (82.8)	384 (78.05)	108 (21.95)
Number of children			
2 or less	441 (73.6)	340 (77.1)	101 (22.9)
3 or more	158 (26.4)	136 (86.08)	22 (13.92)
Child age			
6 months to 1 year	212 (35.4)	140 (66.04)	72 (33.96)
1 year to 1 year and 6 months	203 (33.9)	178 (87.68)	25 (12.32)
1 year and 6 months to 2 years	184 (30.7)	158 (85.87)	26 (14.13)
Child sex			
Female	304 (50.7)	239 (78.62)	65 (21.38)
Male	295 (49.3)	237 (80.34)	58 (19.66)
Monthly household income*			
2 minimum wages or less	432 (72.5)	356 (82.41)	76 (17.59)
2 minimum wages or more	164 (27.5)	117 (71.34)	47 (28.66)
Number of people in the household			
≤4	364 (60.8)	275 (75.55)	89 (24.45)
>4	235 (39.2)	201 (85.53)	34 (14.47)
Home ownership			
Owned	183 (30.6)	142 (77.6)	41 (22.4)
Not owned	416 (69.4)	334 (80.29)	82 (19.71)
Maternal level of education			
≤Incomplete secondary education	539 (90.0)	435 (80.71)	104 (19.29)
>Complete secondary education	60 (10.0)	41 (68.33)	19 (31.67)
Mother works outside			
Yes	193 (32.2)	145 (75.13)	48 (24.87)
No	406 (67.8)	331 (81.53)	75 (18.47)
Household with TV/internet			
Has TV or internet	294 (49.1)	240 (81.63)	54 (18.37)
Has TV and internet	297 (49.6)	229 (77.1)	68 (22.9)
None	8 (1.3)	7 (87.5)	1 (12.5)
Government benefit			
Yes	191 (32.3)	164 (85.86)	27 (14.14)
No	400 (67.7)	304 (76.0)	96 (24.0)

*Minimum wage in 2017 (R$ 937,00).

With regard to maternal socioeconomic characteristics, 52.1% were aged 27 years or under, 82.8% were married/others, 73.6% had two children or less, 72.5% earned two minimum wages or less, 60.8% shared the house with four people or less, 30.6% owned their homes, 90% had incomplete secondary education, 49.6% had access to television and internet, and 32.3% received government benefits (Table 1).

Overall, children aged 1 year to 1 year and six months (87.68%) born from single mothers (87.25%), who had three or more children (86.08%), earned two minimum wages or less (82.41%), shared the house with four people or less (85.53%), had incomplete secondary education (80.71%), and received government benefits (85.86%) were allowed to consume UPF.

Table 2 shows associations (crude and adjusted OR) between UPF consumption and study variables. The following factors were associated with UPF consumption: maternal marital status, monthly household income, child age, number of children and people in the house, maternal level of education, and government benefit. In the adjusted analysis, children aged 1 year to 1 year and six months, and 1 year and six months to 2 years were more likely (OR=3.89; 95%CI: 2.32-6.50; p<0.0001, and OR=3.33; 95%CI: 2.00-5.56; p<0.0001, respectively) to consume UPF relative to children aged 6 months to 1 year. Children living with four people or more were also 1.94-fold more likely (95%CI: 1.23-3.05; p=0.0041) to consume UPF. Children from families granted government benefits were 1.88 times more likely (95%CI: 1.15-3.04; p=0.0112) to consume UPF relative to children from families which were not (Table 2).

**Table 2:** Crude and adjusted odds ratios of ultra-processed food consumption and analyzed variables

Variables	Crude OR	95%CI	p value	Adjusted OR	95%CI	p value
Maternal age, years						
>27	Reference					
≤27	1.14	0.76-1.69	0.6033			
Maternal marital status						
Married/others	Reference					
Single	1.92	1.03-3.58	0.0493			
Number of children						
2 or less	Reference					
3 or more	1.83	1.11-3.03	0.0225			
Child age						
6 months to 1 year	Reference			Reference		
1 year to 1 year and 6 months	3.66	2.21-6.07	<0.0001	3.89	2.32-6.50	<0.0001
1 year and 6 months to 2 years	3.13	1.89-5.17	<0.0001	3.33	2.00-5.56	<0.0001
Child sex						
Female	Reference					
Male	1.11	0.74-1.65	0.6745			
Monthly household income*						
More than R$ 1.874,00	Reference					
R$ 1.874,00 or less	1.88	1.23-2.86	0.0041			
Number of people in the household						
≤4	Reference			Reference		
>4	1.91	1.23-2.95	0.0044	1.94	1.23-3.05	0.0041
Home ownership						
Owned	Reference					
Not owned	1.17	0.77-1.79	0.5211			
Maternal level of education						
≤Incomplete secondary education	Reference					
>Complete secondary education	1.93	1.08-3.47	0.0373			
Mother works outside						
Yes	Reference					
No	1.46	0.96-2.20	0.0885			
Household TV/internet						
Has TV or internet	Reference					
Has TV and internet	1.32	0.88-1.97	0.2083			
None	2.09	0.25-17.19	0.7908			
Government benefit						
No	Reference			Reference		
Yes	1.91	1.20-3.06	0.0079	1.88	1.15-3.04	0.0112

*Minimum wage in 2017 (R$ 937,00).

OR: odds ratio; 95%CI: 95% confidence interval.

## DISCUSSION

Early introduction of ultra-processed foods and insufficient consumption of *in natura* or minimally processed foods may have negative impacts on child health.^(14,15)^

In this sample, 79.4% of children aged 1 to 2 years consumed UPF of some kind. Consumption of such foods was associated with socioeconomic factors.

In Brazil, *in natura* or minimally processed foods tend to be replaced with UPF, with potential health compromise.^(9,16)^ In this study, children aged 1 to 2 years were more likely to consume UPF.

The introduction of UPF is in keeping with studies investigating child feeding, which reported earlier and progressive exposure to unhealthy food consumption according to the age of introducing complementary feeding.^(17)^ Other studies have shown that, at the age of 1 year, children are more exposed to UPF and hence to the development of chronic noncommunicable diseases.^(9,14,18)^

As to socioeconomic variables, children living with four or more people in families receiving *Programa Bolsa Família* (PBF) were more likely to consume UPF. Monthly income below two minimum wages, households with four people, and receiving government benefits are associated with introduction of UPF in the diet of children aged 17 to 63 months.^(19)^ Low monthly income is also a significant factor for introduction of UPF among children aged 4 to 24 months.^(9)^

Lower monthly income, large numbers of family members, parents or responsible persons with lower levels of education, and poor basic sanitation are family profiles associated with food and nutrition insecurity.^(20)^*Programa Bolsa Família* was created by the federal government to combat hunger in the country, via direct transfer of income to poor and extremely poor families. Studies have shown the money received is used to purchase food in most cases, increasing access to food and improving dietary variety.^(21,22)^

Lower UPF consumption among PBF beneficiaries has been reported in the North and Northeast of the country, as well as greater consumption of *in natura* and minimally processed food in the Northeast.^(23)^ In contrast, families living in the city of Curitiba choose low cost, higher energy density foods. Foods *in natura* are not part of the diet in these families, in which higher nutritional density and potential dietary monotony prevail.^(24)^ National research data support greater UPF consumption in regions with greater economic development, such as the South and Southeast.^(25)^

The Ministry of Health launched the *Estratégia Nacional para a Alimentação Complementar Saudável* (ENPACS) [National Strategy for Healthy Complementary Diet] to encourage appropriate complementary feeding guidance provision at health care services, while respecting local dietary habits, in an effort to promote healthy eating habits.^(26)^

In 2012, *Estratégia Amamenta e Alimenta Brasil* [Strategy Breastfeed and Feed Brazil],^(2)^ a joint initiative between ENPACS and *Rede Amamenta Brasil*, was launched to encourage breast feeding and appropriate complementary feeding among infants listed as SUS users.^(27)^ Health units are vital for detecting epidemiologic and nutritional indicators due to their knowledge of respective catchment areas, and may contribute to the development of actions and public policies aimed to encourage appropriate nutrition in each life cycle.^(28)^

Along with strategies designed to promote appropriate and timely introduction of complementary feeding, to reduce UPF consumption, the need to capacitate health professionals must be emphasized. These professionals will be in direct contact with the families of children receiving complementary feeding and must be duly trained to contribute to food and nutritional safety, as well as fulfillment of rights to appropriate feeding.^(29)^ The medical care-centered model of mother and child care, and the lack of appropriate professional training are obstacles to the implementation and continuity of strategies aimed to encourage healthy eating among children.^(30)^

This study may support the planning of health actions to promote increased awareness about the importance of healthy dietary habits among health professionals and families. Inappropriate dietary habits at in early infancy may translate into problems for children in the short- and long-run.^(15,30)^ Hence the need of follow-up, by means of scientific studies, to support ongoing improvements in this important phase of life.

Use of two different data collection instruments may have been a source of inconsistency in responses provided by mothers and is a potential limitation of this study.

## CONCLUSION

This study revealed concerning levels of ultra-processed food consumption among children, particularly those aged 1 to 2 years, and living with more than four people in families granted government benefits. Therefore, sociodemographic and demographic factors play a relevant role in ultra-processed food consumption during complementary feeding.

Specific promotion and preventive actions in nutrition for health professional teams and managers working in vulnerable are needed. Such actions may help educate the population about types of foods and related consequences, and encourage the adoption of a more appropriate and healthy diet, with lower levels of ultra-processed foods.
