# Supplementary material for: Ultra-processed foods consumption among children and associated socioeconomic and demographic factors
Source: Einstein (Sao Paulo). 2021 Aug 25;19:eAO5554. doi: 10.31744/einstein_journal/2021AO5554 (PMC8386687; doi:10.31744/einstein_journal/2021AO5554)
Supplement: Translation

# Translation: Consumo de alimentos ultraprocessados por crianças e fatores socioeconômicos e demográficos associados

**Authors:** Eveline Costa Cainelli, Brunna Verna Castro Gondinho, Danielle da Costa Palacio, Daniele Boina de Oliveira, Roberta Andrade Reis, Karine Laura Cortellazzi, Luciane Miranda Guerra, Denise de Fátima Barros Cavalcante, Antonio Carlos Pereira, Jaqueline Vilela Bulgareli

**Affiliations:** 1 Brasil Faculdade de Odontologia de Piracicaba, Universidade Estadual de Campinas, Piracicaba, SP, Brasil.; 2 Brasil Hospital Israelita Albert Einstein, São Paulo, SP, Brasil.

**Keywords:** Fenômenos fisiológicos da nutrição do lactente, Lactente, Nutrição do lactente, Estratégia saúde da família

## Abstract

**Objetivo:**

Avaliar a ingestão de alimentos ultraprocessados em crianças, e verificar se há associação com o contexto socioeconômico e demográfico.

**Métodos:**

Trata-se de estudo analítico, do tipo transversal, com 599 crianças entre 6 meses e 2 anos de idade, cadastradas em Unidades de Saúde da Família, de um município de médio porte. Para a realização da coleta, as mães das crianças foram abordadas em seus domicílios pelas pesquisadoras e por um Agente Comunitário de Saúde da Unidade Saúde da Família e responderam dois questionários, o socioeconômico e demográfico e o marcador do Sistema de Vigilância Alimentar e Nutricional do Ministério da Saúde do Brasil para crianças entre 6 meses e 2 anos. A variável dependente do estudo foi a ingestão de alimentos ultraprocessados pela criança e as independentes foram as socioeconômicas e demográficas. Foi realizada análise de regressão múltipla, no nível de significância de 5%, para testar a associação entre a ingestão de alimentos ultraprocessados com as variáveis socioeconômicas e demográficas.

**Resultados:**

A ingestão de ultraprocessados esteve associada com a idade da criança entre 1 e 2 anos (RC=3,89; IC95%: 2,32-6,50 e RC=3,33; IC95%: 2,00-5,56, respectivamente), com o número de pessoas que residiam na mesma casa (RC=1,94; IC95%: 1,23-3,05) e com as famílias que recebiam auxílio do governo (RC=1,88; IC95%: 1,15-3,04).

**Conclusão:**

A ingestão de alimentos ultraprocessados por crianças no período da alimentação complementar pode ser influenciada por fatores socioeconômicos e demográficos.

## INTRODUÇÃO

Os primeiros 1.000 dias de vida definem o ciclo que vai desde a fecundação até os 2 primeiros anos de idade, tendo importância direta no desenvolvimento humano. Do ponto de vista nutricional, a suplementação na gravidez, o aleitamento materno e a alimentação complementar são três importantes estratégias comprovadas com efetividade nesse período.^(1)^

A alimentação complementar é a fase que inicia a partir do sexto mês de vida do bebê, durante os quais novos alimentos amassados ou em forma de purês, provenientes de todos os grupos alimentares, devem ser oferecidos (além do leite materno) de forma lenta e progressiva.^(2)^

A partir de 6 meses, há maior tolerância gastrintestinal e capacidade de absorção de nutrientes, permitindo adaptação física e fisiológica da criança para uma alimentação heterogênea quanto à consistência e à textura. É importante variar os alimentos ofertados e evitar os que sejam ricos em açúcares, gorduras saturadas e trans, aditivos e corantes alimentares, pois, além de as crianças receberem todos os nutrientes necessários, ocorrerá o estímulo para a formação dos hábitos alimentares e a monotonia alimentar será evitada.^(3)^

Os alimentos ultraprocessados (AUP) são conceituados pelas guloseimas, bebidas adoçadas com açúcar ou adoçantes artificiais, embutidos e vários outros que surgem nas prateleiras a cada ano. São formulações industriais feitas inteiramente de substâncias extraídas de alimentos ou sintetizadas em laboratório com base em matérias orgânicas, como o petróleo e o carvão.^(4,5)^

As evidências científicas apontam que a substituição de alimentos caseiros e *in natura* por AUP leva a excesso de peso, doenças crônicas não transmissíveis e carências nutricionais específicas na infância, podendo repercutir na vida adulta.^(6)^ Essas modificações têm sido observadas em todos os níveis socioeconômicos – até mesmo nos de baixa renda.^(7)^

No Brasil, o excesso de peso e a obesidade entre a população se mostram relevantes, especialmente em crianças.^(8)^

Os AUPs devem ser evitados no período de alimentação complementar, pois podem repercutir de forma negativa na saúde geral e bucal da criança.^(9,10)^ Avaliar o consumo de AUP nesse período e se há associação com fatores socioeconômicos e demográficos permitirá uma visão ampliada da escolha desses alimentos pelos pais ou responsáveis da criança, servindo como base para que os profissionais de saúde e gestores repensem as ações e as políticas direcionadas para a melhora da alimentação infantil.

## OBJETIVO

Avaliar a ingestão de alimentos ultraprocessados em crianças e verificar sua associação com fatores socioeconômicos e demográficos.

## MÉTODOS

### Considerações éticas

De acordo com as normas da resolução 466/2012 do Conselho Nacional de Saúde do Ministério da Saúde, o estudo foi aprovado pelo Comitê de Ética em Pesquisa da Faculdade de Odontologia de Piracicaba, Universidade Estadual de Campinas (UNICAMP), parecer 1.852.022, CAAE: 61502116.6.0000.5418. Todos os participantes assinaram o Termo de Consentimento Livre e Esclarecido (TCLE).

### Local, população e tipo do estudo

Estudo realizado em Piracicaba (SP), município considerado de médio porte, contando com população estimada de 404.142 mil habitantes, área territorial de 1.378,069km^2^ e densidade demográfica de 264,47 habitantes/km^2^, região que possui 122 estabelecimentos de saúde do Sistema Único de Saúde (SUS).^(11)^

Trata-se de um estudo analítico e transversal, cuja população-alvo foi formada por crianças de 6 meses a 2 anos cadastradas nas Unidades de Saúde da Família (USF) do município, totalizando 1.169 crianças no período de janeiro a abril de 2016, segundo a Secretaria Municipal de Saúde.

### Seleção e amostra

O tamanho da amostra foi calculado no Epi Info™ 7, considerando nível de intervalo de confiança de 95% (IC95%), poder do teste de 80%, taxa de não expostos/expostos de 1, percentagem de resposta no grupo não exposto de 73% e razão de chance (RC) de 1,8, obtendo-se tamanho mínimo da amostra de 582 indivíduos selecionados aleatoriamente.

Foram incluídas na amostra mães que estavam presentes no dia da coleta de dados, com faixa etária entre 18 a 50 anos de idade, que possuíam filhos entre 6 meses e 2 anos e tinham conhecimento sobre a alimentação do filho no dia anterior.

### Delineamento do estudo

O estudo foi realizado com 599 crianças cadastradas como usuárias nas USFs do município, de fevereiro a julho de 2017. O agendamento de data e horário para que as pesquisadoras pudessem realizar a coleta de dados foi via contato telefônico com gestores das USFs.

O contato com as mães foi realizado em uma fase na qual as pesquisadoras e um Agente Comunitário de Saúde se deslocavam da unidade para o domicílio. As pesquisadoras explicavam a finalidade da pesquisa, e as mães que aceitavam participar assinavam o TCLE e respondiam a questões referentes aos dados socioeconômicos e demográficos e sobre a alimentação do dia anterior do filho. As mães puderam esclarecer dúvidas sobre a alimentação dos filhos.

### Instrumento para coleta de dados

Para a coleta de dados socioeconômicos, foram utilizadas questões baseadas no instrumento de Meneghim et al.,^(10)^ (Apêndice 1), tendo sido adicionadas perguntas referentes a características demográficas (idade, estado civil, número de filhos e se a mãe trabalha fora do lar, quem é o chefe da família, a casa possui televisão e/ou internet e se recebem auxílio do governo).

**Apêndice 1:** Questionário baseado no instrumento de Meneghim et al.,(10)

Questionário socioeconômico e demográfico									
Nome da mãe: _____________________________								Idade: _______	
Nome da criança: ____________________________								Idade: _______	
Sexo da criança:	a) F	b) M		Data de nascimento da criança: ___-___-___					
1. Estado civil:	a) Solteira	b) Casada		c) Vivendo como casado			d) Separada	e) Divorciada	f) Viúva
2. Quantos filhos você tem (sem considerar esta criança):			a) 1		b) 2	c) 3	d) 4	e) More than 5	
3. Situação econômica da família (renda familiar mensal em salários):									
a) Até R$ 937.00	b) De R$ 937.00 a R$ 1,874.00								
c) De R$ 1,874.00 a R$ 2,811.00	d) De R$ 2,811.00 a R$ 4,685.00								
e) De R$ 4,685.00 a R$ 6,559.00	f) De R$ 6,559/00 a R$ 10,307.00								
g) Acima R$ 10,307.00									
4. Qual o número de pessoas que moram na mesma casa:									
a) Até 2 pessoas				b) 3 pessoas				c) 4 pessoas	
d) 5 pessoas				e) 6 pessoas				f) Acima de 6 pessoas	
5. Sua residência é:									
a) Residência própria quitada				b) Residência própria com financiamento a pagar					
c) Residência cedida pelos pais ou parentes				d) Residência cedida em troca de trabalho					
e) Residência alugada				f) Residência cedida por não ter onde morar					
6. Qual é o seu grau de instrução:									
a) Não alfabetizado				b) Alfabetizado					
c) 1ª e 4ª série incompleta				d) 1ª e 4ª série completa					
e) 5ª e 8ª série incompleta				f) 5ª e 8ª série completa					
g) 2° grau incompleto				h) 2° grau completo					
i) Superior incompleto				j) Superior completo					
7. Quem é o chefe da família?									
a) Mãe				b) Pai					
8. Qual a profissão do chefe da família (citar mesmo que desempregado)? __________________________									
9. Você trabalha fora do lar?				a) Sim				b) Não	
10. Se sim, quem fica com a criança no seu horário de trabalho? ___________________________									
11. A casa possui?	a) Televisão			b) Internet				c) Televisão e internet	
12. Recebe auxílio de programas do governo (citar)?				a) Sim _________________				b) Não	

O presente estudo utilizou o formulário Marcadores de Consumo Alimentar do Sistema de Vigilância Alimentar e Nutricional (Sisvan) do Ministério da Saúde para crianças entre 6 meses e 2 anos de idade, baseado em um documento publicado pela Organização Mundial da Saúde (OMS)^(12)^ (Apêndice 2).

**Apêndice 2:** Formulário de consumo alimentar para crianças entre 6 meses e 2 anos de idade

Assinalar com um X todas as perguntas:	( ) Sim	( ) Não	( ) Não sabe			
A criança ontem tomou leite do peito?				( ) Sim	( ) Não	( ) Não sabe
Ontem a criança comeu fruta inteira, em pedaço ou amassada?				( ) Sim	( ) Não	( ) Não sabe
Se sim, quantas vezes?			( ) 1 vez	( ) 2 vezes	( ) 3 vezes ou mais	( ) Não sabe
Ontem a criança comeu comida de sal (de panela, papa ou sopa)?				( ) Sim	( ) Não	( ) Não sabe
Se sim, quantas vezes?			( ) 1 vez	( ) 2 vezes	( ) 3 vezes ou mais	( ) Não sabe
Se sim, essa comida foi oferecida?			( ) Em pedaços	( ) Amassada	( ) Passada na peneira	( ) Liquidificada
Outro leite que não o leite do peito				( ) Sim	( ) Não	( ) Não sabe
Mingau com leite				( ) Sim	( ) Não	( ) Não sabe
Iogurte				( ) Sim	( ) Não	( ) Não sabe
Legumes (não considerar cará, inhame, batata, mandioca)				( ) Sim	( ) Não	( ) Não sabe
Vegetal ou fruta de cor alaranjada (mamão, abobora) ou vegetais verde-escuro (couve, espinafre, almeirão)				( ) Sim	( ) Não	( ) Não sabe
Verdura de folha (alface, acelga, repolho).				( ) Sim	( ) Não	( ) Não sabe
Carne, miúdos ou ovo				( ) Sim	( ) Não	( ) Não sabe
Fígado				( ) Sim	( ) Não	( ) Não sabe
Feijão				( ) Sim	( ) Não	( ) Não sabe
Arroz, batata, inhame, mandioca, farinha ou macarrão (sem ser instantâneo)				( ) Sim	( ) Não	( ) Não sabe
Hambúrguer ou embutidos: salsicha, presunto, salame				( ) Sim	( ) Não	( ) Não sabe
Bebidas adoçadas (suco em pó, refrigerante, suco de caixinha, suco de fruta com açúcar, achocolatados e engrossantes)				( ) Sim	( ) Não	( ) Não sabe
Macarrão instantâneo, salgadinhos de pacote ou biscoito salgado				( ) Sim	( ) Não	( ) Não sabe
Biscoito recheado, doces ou guloseimas (gelatina, bala, pirulito e chiclete)				( ) Sim	( ) Não	( ) Não sabe

O formulário apresenta questões sobre a qualidade e o tempo oportuno na introdução de alimentos, a identificação de risco ou proteção para as carências nutricionais e, também, a ocorrência de sobrepeso. Contém 20 perguntas fechadas, às quais a mãe ou o responsável pela criança responde “sim”, “não” ou “não sabe”. Foram incluídos sexo e idade da criança e coletadas todas as 20 perguntas referentes ao marcador, mas só a categoria de AUP foi utiliza na pesquisa.

### Variáveis do estudo

A variável dependente deste estudo foi a ingestão de AUP, sendo dicotomizada em sim e não. Foram considerados AUP: hambúrguer e ou embutidos (presunto, mortadela, salame, linguiça e salsicha); bebidas adoçadas (refrigerante, suco de caixinha, suco em pó, água de coco de caixinha, xaropes de guaraná ou groselha e suco de fruta com adição de açúcar); macarrão instantâneo, salgadinhos de pacote ou biscoitos salgados e biscoito recheado, doces ou guloseimas, conforme o marcador.

As variáveis independentes foram idade, estado civil e número de filhos da mãe, renda familiar, número de pessoas na casa, tipo de residência, nível de escolaridade materna e se a casa possuía televisão e/ou internet dicotomizadas pela mediana; as variáveis sexo da criança foram classificadas como feminino ou masculino e chefe da família como mãe ou pai. A idade da criança foi dividida por categorias: 6 meses a 1 ano, 1 ano a 1 ano e 6 meses e 1 ano e 6 meses a 2 anos, de acordo com as orientações sugeridas pelo marcador.^(13)^

### Análise dos dados

Para avaliar a associação entre a ingestão de AUP e as variáveis independentes, aplicou-se análise de regressão logística múltipla. Foram testadas no modelo de regressão logística múltipla as variáveis com p≤0,20 na análise bruta, permanecendo no modelo múltiplo aquelas que continuaram associadas à ingestão de AUP com p≤0,05 após o ajuste para as demais variáveis analisadas. As RC e os respectivos IC95% foram estimados. O nível de significância adotado foi de 5%. Todos os testes estatísticos foram realizados pelo programa SAS, versão 9.4 (SAS Institute Inc., Cary, NC, Estados Unidos, release 9.4, 2010).

## RESULTADOS

A tabela 1 mostra a distribuição de frequências da ingestão de AUP em função das variáveis analisadas. A ingestão de AUP foi constatada em 79,4% das crianças, sendo 50,7% do sexo feminino, 35,4% com idade entre 6 meses e 1 ano, 33,9% com 1 ano a 1 ano e 6 meses e 30,7% com 1 ano e 6 meses a 2 anos.

**Tabela 1:** Ingestão de alimentos ultraprocessados em função das variáveis

Variável	Consumo de alimentos ultraprocessados		
	Total	Sim	Não
Idade da mãe, anos			
≤27	312 (52,1)	251 (80,45)	61 (19,55)
>27	287 (47,9)	225 (78,4)	62 (21,6)
Estado civil da mãe			
Solteira	102 (17,2)	89 (87,25)	13 (12,75)
Casada/outros	492 (82,8)	384 (78,05)	108 (21,95)
Número de filhos			
Até 2	441 (73,6)	340 (77,1)	101 (22,9)
3 ou mais	158 (26,4)	136 (86,08)	22 (13,92)
Idade da criança			
6 meses a 1 ano	212 (35,4)	140 (66,04)	72 (33,96)
1 ano a 1 ano e 6 meses	203 (33,9)	178 (87,68)	25 (12,32)
1 ano e 6 meses a 2 anos	184 (30,7)	158 (85,87)	26 (14,13)
Sexo da criança			
Feminino	304 (50,7)	239 (78,62)	65 (21,38)
Masculino	295 (49,3)	237 (80,34)	58 (19,66)
Renda familiar mensal*			
Até 2 salários mínimos	432 (72,5)	356 (82,41)	76 (17,59)
Acima de 2 salários mínimos	164 (27,5)	117 (71,34)	47 (28,66)
Número de pessoas na casa			
≤4	364 (60,8)	275 (75,55)	89 (24,45)
>4	235 (39,2)	201 (85,53)	34 (14,47)
Tipo de residência			
Própria	183 (30,6)	142 (77,6)	41 (22,4)
Não própria	416 (69,4)	334 (80,29)	82 (19,71)
Escolaridade da mãe			
≤2º grau completo	539 (90,0)	435 (80,71)	104 (19,29)
>2º grau completo	60 (10,0)	41 (68,33)	19 (31,67)
Mãe trabalha fora			
Sim	193 (32,2)	145 (75,13)	48 (24,87)
Não	406 (67,8)	331 (81,53)	75 (18,47)
Na casa tem TV/internet			
Tem TV ou internet	294 (49,1)	240 (81,63)	54 (18,37)
TV e internet	297 (49,6)	229 (77,1)	68 (22,9)
Nenhum	8 (1,3)	7 (87,5)	1 (12,5)
Recebe auxílio do governo			
Sim	191 (32,3)	164 (85,86)	27 (14,14)
Não	400 (67,7)	304 (76,0)	96 (24,0)

* Salário mínimo referente ao ano de 2017 (R$ 937,00).

Em relação às características socioeconômicas da mãe, 52,1% tinham idade menor ou igual a 27 anos, 82,8% eram casadas/outros, 73,6% tinham até dois filhos, 72,5% tinham renda mensal de até dois salários mínimos, 60,8% moravam com quatro pessoas ou menos, 30,6% tinham residência própria, 90% com ≤2º grau completo, 49,6% possuíam acesso à televisão e à internet e 32,3% recebiam auxílio do governo (Tabela 1).

Do total, as crianças com idade entre 1 e 1 ano e 6 meses (87,68%), cujas mães eram solteiras (87,25%), com três ou mais filhos (86,08%), com renda até dois salários mínimos (82,41%), residiam com mais de quatro pessoas (85,53%), com ≤2º grau completo (80,71%) e recebiam auxílio do governo (85,86%) permitiam que os filhos ingerissem AUP.

A tabela 2 apresenta as RC brutas e ajustadas entre a ingestão de AUP com as variáveis analisadas. Os fatores associados com a ingestão de AUP foram estado civil da mãe, renda familiar mensal, idade da criança, número de filhos e de pessoas na casa, escolaridade da mãe e auxílio governo. Na análise ajustada, as crianças com idade entre 1 ano e 1 ano e 6 meses, e 1 ano e 6 meses e 2 anos tiveram maior chance (RC=3,89; IC95%: 2,32-6,50; p<0,0001 e RC=3,33; IC95%: 2,00-5,56; p<0,0001, respectivamente) de ingerir AUP quando comparadas às de 6 meses a 1 ano. Sobre o número de pessoas na família, crianças que residiam com mais de quatro pessoas tiveram 1,94 maior chance (IC95%: 1,23-3,05; p=0,0041) de ingerir AUP. Os filhos de famílias que recebiam auxílio do governo tiveram 1,88 vez mais chance (IC95%: 1,15-3,04; p=0,0112) de ingerir AUP do que aqueles que não recebiam (Tabela 2).

**Tabela 2:** Razão de chance brutos e ajustados entre a ingestão de alimentos ultraprocessados e as variáveis analisadas

Variáveis	RC bruta	IC95%	Valor de p	RC ajustada	IC95%	Valor de p
Idade da mãe, anos						
>27	Referência					
≤27	1,14	0,76-1,69	0,6033			
Estado civil da mãe						
Casada/outros	Referência					
Solteiro	1,92	1,03-3,58	0,0493			
Número de filhos						
Até 2	Referência					
3 ou mais	1,83	1,11-3,03	0,0225			
Idade da criança						
6 meses a 1 ano	Referência			Referência		
1 ano a 1 ano e 6 meses	3,66	2,21-6,07	<0,0001	3,89	2,32-6,50	<0,0001
1 ano e 6 meses a 2 anos	3,13	1,89-5,17	<0,0001	3,33	2,00-5,56	<0,0001
Sexo da criança						
Feminino	Referência					
Masculino	1,11	0,74-1,65	0,6745			
Renda familiar mensal*						
Acima de R$ 1.874,00	Referência					
Até R$ 1.874,00	1,88	1,23-2,86	0,0041			
Número de pessoas na casa						
≤4	Referência			Referência		
>4	1,91	1,23-2,95	0,0044	1,94	1,23-3,05	0,0041
Tipo de residência						
Própria	Referência					
Não própria	1,17	0,77-1,79	0,5211			
Escolaridade da mãe						
>2º grau completo	Referência					
≤2º grau completo	1,93	1,08-3,47	0,0373			
Mãe trabalha fora						
Sim	Referência					
Não	1,46	0,96-2,20	0,0885			
Na casa tem TV/internet						
TV e internet	Referência					
Tem TV ou internet	1,32	0,88-1,97	0,2083			
Nenhum	2,09	0,25-17,19	0,7908			
Recebe auxílio do governo						
Não	Referência			Referência		
Sim	1,91	1,20-3,06	0,0079	1,88	1,15-3,04	0,0112

* Salário mínimo referente ao ano de 2017 (R$ 937,00).

RC: razão de chance; IC95%: intervalo de confiança de 95%.

## DISCUSSÃO

Alimentos ultraprocessados, quando oferecidos precocemente na alimentação infantil, e ingestão inoportuna de alimentos *in natura* ou minimamente processados podem desencadear alterações negativas na saúde da criança.^(14,15)^

Foi possível observar que 79,4% das crianças de 1 a 2 anos de idade ingeriram algum tipo de AUP. As características socioeconômicas se apresentaram como condições relevantes para a ingestão desses alimentos.

Atualmente, a tendência brasileira é substituir alimentos *in natura* ou minimamente processados por AUP, podendo desenvolver agravos para a saúde.^(9,16)^ No presente estudo, as crianças com idade entre 1 e 2 anos tiveram mais chance de ingerir AUP.

A introdução desses alimentos corrobora estudos sobre o consumo alimentar infantil, os quais demonstram maior exposição à ingestão de alimentos não saudáveis, de maneira precoce e crescente segundo a faixa etária da alimentação complementar.^(17)^ Outros estudos apontam que as crianças, ao completarem o primeiro ano de vida, estão mais expostas aos AUP e, portanto, ao desenvolvimento de doenças crônicas não transmissíveis.^(9,14,18)^

Em relação às variáveis socioeconômicas, as crianças que residiam com mais de quatro pessoas e recebiam o Programa Bolsa Família (PBF) tiveram maiores chances de ingerir AUP. Renda mensal menor que dois salários mínimos, famílias com quatro moradores no domicílio e recebimento de alguma forma de benefício do governo são associados com a introdução de AUP na alimentação de crianças entre 17 a 63 meses.^(19)^ Além disso, a baixa renda mensal é fator significativo para a introdução de AUP em crianças de 4 a 24 meses.^(9)^

Menor renda mensal, maior número de membros, responsáveis com menor escolaridade e saneamento básico precários são perfis familiares de insegurança alimentar e nutricional.^(20)^ O PBF foi criado pelo governo federal para combater a fome no país por meio da transferência direta de renda às famílias pobres e extremamente pobres. Estudos identificam que o dinheiro repassado é, na maioria das vezes, utilizado para a compra de alimentos, garantindo o acesso e uma maior variedade de alimentos.^(21,22)^

Os beneficiários do PBF nas regiões Norte e Nordeste apresentam consumo menor de AUP e maior de alimentos *in natura* e minimamente processados na região Nordeste.^(23)^ Em contrapartida, em Curitiba, optam por alimentos de baixo custo e com maior densidade energética. Alimentos *in natura* não fazem parte da alimentação dessas famílias, caracterizando-as com maior densidade nutricional e possível monotonia alimentar.^(24)^ Pesquisa em âmbito nacional confirma que o consumo de AUP é maior em regiões economicamente mais desenvolvidas, como Sul e Sudeste.^(25)^

O Ministério da Saúde lançou a Estratégia Nacional para a Alimentação Complementar Saudável (ENPACS), com a finalidade de estimular a orientação adequada da alimentação complementar nos serviços de saúde, respeitando os hábitos locais nas regiões do país, de forma a desenvolver hábitos alimentares saudáveis.^(26)^

Em 2012, foi criada a Estratégia Amamenta e Alimenta Brasil,^(2)^ que foi uma junção da ENPACS com a Rede Amamenta Brasil, objetivando promover o aleitamento materno e a alimentação complementar adequada dos lactentes no SUS.^(27)^ As unidades de saúde são primordiais para a detecção de indicadores epidemiológicos e nutricionais por meio do conhecimento do território de abrangência, contribuindo para ações e desenvolvimento de políticas públicas que visam estimular a nutrição adequada em cada ciclo da vida.^(28)^

Além da importância de estratégias direcionadas a uma alimentação complementar adequada, oportuna, visando à redução da ingestão de AUP, vale ressaltar a importância da capacitação dos profissionais que atenderão as famílias com crianças no período da alimentação complementar, para que eles contribuam com mecanismos capazes de redundarem na segurança alimentar e nutricional e no direito à alimentação adequada.^(29)^ O modelo centrado na atuação médica em relação ao binômio mãe e bebê e as condições profissionais inadequadas dificultam a realização e a continuidade de estratégias desenvolvidas para a promoção da alimentação infantil saudável.^(30)^

Esta pesquisa pode servir como ferramenta para o planejamento de ações de saúde voltadas para a conscientização dos profissionais de saúde e das famílias sobre a importância dos hábitos alimentares saudáveis. Os hábitos alimentares inadequados na primeira infância podem desencadear problemas a curto e longo prazo para as crianças.^(15,30)^ Por isso, a necessidade de acompanhamento por meio de estudos científicos é válida para que haja constância de melhora nesse período tão importante da vida.

Por outro lado, o estudo teve limitações nas respostas das mães em dois instrumentos diferentes, o que poderia dar a chance de ocorrer alguma irregularidade nas respostas.

## CONCLUSÃO

Este estudo constatou uma ingestão preocupante de alimentos ultraprocessados pelas crianças, principalmente as com idade entre 1 e 2 anos, que residiam com mais de quatro pessoas e que estavam inseridas em famílias que recebiam auxílio do governo. Dessa forma, observou-se que a ingestão de ultraprocessados no período da alimentação complementar, pode ser influenciada por fatores socioeconômicos e demográficos.

São necessárias ações pontuais de promoção e prevenção em nutrição para equipes de saúde e gestores que atuam em áreas de vulnerabilidade. Dessa forma, a população terá mais chances de compreender os vários tipos de alimentos existentes e suas consequências, possibilitando uma alimentação adequada e saudável, com menor ingestão de ultraprocessados.
